# Concern regarding the alleged spread of hypervirulent lymphogranuloma venereum *Chlamydia trachomatis* strain in Europe

**DOI:** 10.2807/1560-7917.ES.2017.22.15.30511

**Published:** 2017-04-13

**Authors:** Helena MB Seth-Smith, Juan C Galán, Daniel Goldenberger, David A Lewis, Olivia Peuchant, Cecile Bébéar, Bertille de Barbeyrac, Angele Bénard, Ian Carter, Jen Kok, Sylvia M Bruisten, Bart Versteeg, Servaas A Morré, Nicholas R Thomson, Adrian Egli, Henry JC de Vries

**Affiliations:** 1 Clinical Microbiology, University Hospital Basel, Basel, Switzerland; 2 Applied Microbiology Research, Department of Biomedicine, University of Basel, Basel, Switzerland; 3 Servicio de Microbiología, Hospital Universitario Ramón y Cajal. CIBER en Epidemiología y Salud Pública (CIBERESP). Instituto Ramón y Cajal de Investigación Sanitaria (IRYCIS), Madrid, Spain; 4 Western Sydney Sexual Health Centre, Western Sydney Local Health District, Parramatta, New South Wales, Australia; 5 Marie Bashir Institute for Infectious Diseases and Biosecurity & Sydney Medical School-Westmead, Sydney, Australia; 6 University of Bordeaux, INRA, USC ES 3671, French National Reference Centre for chlamydiae, Bordeaux, France; 7 Wellcome Trust Sanger Institute. Cambridge, United Kingdom; 8 Centre for Infectious Diseases and Microbiology Laboratory Services, Institute of Clinical Pathology and Medical Research, Westmead Hospital, Westmead, New South Wales, Australia; 9 STI Outpatient Clinic, Department of Infectious Diseases, Public Health Service Amsterdam, Amsterdam, the Netherlands; 10 Amsterdam Infection and Immunity Institute (AI&II), Academic Medical Centre, University of Amsterdam, Amsterdam, the Netherlands; 11 Laboratory of Immunogenetics, Department of Medical Microbiology and Infection Control, VU University Medical Center Amsterdam, Amsterdam, the Netherlands; 12 Institute for Public Health Genomics (IPHG), Department of Genetics and Cell Biology, Research Institute GROW, University of Maastricht, Maastricht, the Netherlands; 13 London School of Hygiene and Tropical Medicine, London, United Kingdom; 14 Department of Dermatology, Academic Medical Center, University of Amsterdam, Amsterdam, the Netherlands

**Keywords:** lymphogranuloma venereum, *Chlamydia trachomatis*, male homosexuality, bacterial outer membrane proteins, genotype

**To the editor:** A recent surveillance and outbreak report published in *Eurosurveillance* by Petrovay et al. on the ‘Emergence of the lymphogranuloma venereum L2c genovariant, Hungary, 2012 to 2016’ [1] provides an observation of the first European cases of a genotype of *Chlamydia trachomatis* associated with severe haemorrhagic proctitis. The authors of this paper diagnosed the strains as lymphogranuloma venereum (LGV)-associated and performed partial sequencing of the *ompA* gene (ca 1,070 bp), which is a standard typing method for *C. trachomatis*. The *ompA* gene sequence obtained was compared with those from reference isolates, and reported to be 100% concordant with the *ompA* sequence belonging to an L2-D recombinant strain described in 2011 [2]. This strain was named ‘L2c’, as it was found to possess a chimeric genome, not because it has a novel *ompA*-genotype. We would like to point out that the *ompA* gene sequence of this L2-D recombinant strain, and by implication those of the Hungarian isolates, is identical to that of archetypal L2 strains, for example the reference strain L2/434 [3].

Petrovay et al. found that the *pmpH*-genotype of the Hungarian strains reflect that of an LGV strain, containing a diagnostic 36 bp deletion. Unfortunately this locus does not discriminate between L2 strains and L2-D. As the authors appear not to have checked for concordance between their strains and the L2-D recombinant strain in other genomic loci, it is not possible to determine whether the strains reflect the appearance of this L2-D recombinant, or rather a circulating L2 LGV strain. Thus, it is premature to assume that these Hungarian LGV strains reflect the presence of the ‘hypervirulent’ L2-D recombinant strain, despite the described clinical symptoms. We find it more likely that the authors have observed a resurgence in cases with *ompA*-genotype L2, as described last year [4].

For the *Chlamydia* community, it is important to recognise that the use of the term ‘L2c genotype’ in the case of the L2-D recombinant strain is a misnomer, as the *ompA*-genotype of this strain is an archetypal L2. This nomenclature was also the source of confusion in a recent paper from Slovenia describing the presence of ‘L2c’ [5], again with further analysis now showing that the *ompA*-genotype of this strain is also identical to L2. The distinct L2c *ompA*-genotype was described in a 2008 publication, and has 2 nucleotide differences to that of L2 [6].

Given the high level of recombination observed in *C. trachomatis* [7], typing techniques based on a few loci can never give a full indication of the underlying genomic background: only whole genome sequencing and detailed phylogenetic analysis can provide this. Therefore we would recommend that future publications are absolutely clear as to which genotyping method they have used in strain descriptions, for example a common target such as the *ompA*-genotype. Furthermore, *Chlamydia* researchers should be aware of this awkwardness of nomenclature, should thoroughly compare their *ompA* sequences against a database of known L2 *ompA*-genotypes (L2: AM884176; L2a: AB915594, L2b: AM884177; L2c: EF460796; L2d: EF460797; L2e: EF460798; L2f: EU676181; L2g: EU676180; L2bV1: JX971936; L2bV2: KU518893; L2bV3: KU518894; L2bV4: KU518892) [3,6,8-10], and report their findings more fully.

As it stands, the description of the Hungarian strains as ‘L2c’ is inaccurate in the sense of the *ompA*-genotype. Importantly, it is not possible to make any conclusions about the European appearance of this ‘hypervirulent’ L2-D recombinant strain without further sequencing of additional genomic loci, ideally whole genome sequencing, or investigations into in vitro phenotypes.
